# Duration of Untreated Psychosis in First-Episode Psychosis is not Associated With Common Genetic Variants for Major Psychiatric Conditions: Results From the Multi-Center EU-GEI Study

**DOI:** 10.1093/schbul/sbab055

**Published:** 2021-05-08

**Authors:** Olesya Ajnakina, Victoria Rodriguez, Diego Quattrone, Marta di Forti, Evangelos Vassos, Celso Arango, Domenico Berardi, Miguel Bernardo, Julio Bobes, Lieuwe de Haan, Cristina Marta Del-Ben, Charlotte Gayer-Anderson, Hannah E Jongsma, Antonio Lasalvia, Sarah Tosato, Pierre-Michel Llorca, Paulo Rossi Menezes, Bart P Rutten, Jose Luis Santos, Julio Sanjuán, Jean-Paul Selten, Andrei Szöke, Ilaria Tarricone, Giuseppe D’Andrea, Alexander Richards, Andrea Tortelli, Eva Velthorst, Peter B Jones, Manuel Arrojo Romero, Caterina La Cascia, James B Kirkbride, Jim van Os, Mick O’Donovan, Robin M Murray, EU-GEI WP2 Group

**Affiliations:** 1 Department of Biostatistics & Health Informatics, Institute of Psychiatry, Psychology and Neuroscience, King’s College London, University of London, London, UK; 2 Department of Behavioural Science and Health, Institute of Epidemiology and Health Care, University College London, London, UK; 3 Department of Clinical Medicine, Aarhus University, Aarhus, Denmark; 4 Department of Psychosis Studies, Institute of Psychiatry, Psychology and Neuroscience, King’s College London, London, UK; 5 Social, Genetic and Developmental Psychiatry Centre, Institute of Psychiatry, Psychology and Neuroscience, King’s College London, London, UK; 6 Child and Adolescent Psychiatry Department, Institute of Psychiatry and Mental Health, Hospital General Universitario Gregorio Marañón, School of Medicine, Universidad Complutense, Madrid, Spain; 7 Department of Biomedical and Neuromotor Sciences, Psychiatry Unit, Alma Mater Studiorum Università di Bologna, Bologna, Italy; 8 Department of Psychiatry, Barcelona Clinic Schizophrenia Unit, Neuroscience Institute, Hospital Clinic of Barcelona, University of Barcelona, IDIBAPS, CIBERSAM, Barcelona, Spain; 9 Faculty of Medicine and Health Sciences – Psychiatry, Universidad de Oviedo, ISPA, INEUROPA, CIBERSAM, Oviedo, Spain; 10 Department of Psychiatry, Early Psychosis Section, Amsterdam UMC, University of Amsterdam, Amsterdam, The Netherlands; 11 Neuroscience and Behavior Department, Ribeirão Preto Medical School, University of São Paulo, São Paulo, Brazil; 12 Department of Health Service and Population Research, Institute of Psychiatry, Psychology and Neuroscience, King’s College London, De Crespigny Park, Denmark Hill, London, UK; 13 Centre for Longitudinal Studies, University College London, London, UK; 14 Centre for Transcultural Psychiatry Veldzicht, Balkbrug, The Netherlands; 15 University Centre for Psychiatry, University Medical Centre Groningen, Groningen, The Netherlands; 16 Section of Psychiatry, Department of Neuroscience, Biomedicine and Movement Sciences, University of Verona, Verona, Italy; 17 Université Clermont Auvergne, CMP-B CHU, CNRS, Clermont Auvergne INP, Institut Pascal, Clermont-Ferrand, France; 18 Department of Preventative Medicine, Faculdade de Medicina FMUSP, University of São Paulo, São Paulo, Brazil; 19 Department of Psychiatry and Neuropsychology, School for Mental Health and Neuroscience, South Limburg Mental Health Research and Teaching Network, Maastricht University Medical Centre, Maastricht, The Netherlands; 20 Department of Psychiatry, Servicio de Psiquiatría Hospital “Virgen de la Luz,” Cuenca, Spain; 21 Department of Psychiatry, Hospital Clínico Universitario de Valencia, INCLIVA, CIBERSAM, School of Medicine, Universidad de Valencia, Valencia, Spain; 22 Rivierduinen Institute for Mental Health Care, Sandifortdreef 19, 2333 ZZ Leiden, The Netherlands; 23 Univ Paris Est Creteil, INSERM, IMRB, AP-HP, Hôpitaux Universitaires “ H. Mondor ,” DMU IMPACT, Fondation FondaMental, Creteil, France; 24 Division of Psychological Medicine and Clinical Neurosciences, Cardiff, UK; 25 Etablissement Public de Santé Maison Blanche, Paris, France; 26 Department of Psychiatry, Early Psychosis Section, Academic Medical Centre, University of Amsterdam, Amsterdam, The Netherlands; 27 Department of Psychiatry, Icahn School of Medicine at Mount Sinai, New York, NY; 28 Department of Psychiatry, University of Cambridge, Herchel Smith Building for Brain & Mind Sciences, Cambridge, UK; 29 CAMEO Early Intervention Service, Cambridgeshire & Peterborough NHS Foundation Trust, Cambridge, UK; 30 Department of Psychiatry, Psychiatric Genetic Group, Instituto de Investigación Sanitaria de Santiago de Compostela, Complejo Hospitalario Universitario de Santiago de Compostela, Santiago de Compostela, Spain; 31 Department of Experimental Biomedicine and Clinical Neuroscience, University of Palermo, Palermo, Italy; 32 Psylife Group, Division of Psychiatry, University College London, London, UK; 33 Department of Psychiatry and Neuropsychology, School for Mental Health and Neuroscience, South Limburg Mental Health Research and Teaching Network, Maastricht University Medical Centre, Maastricht, The Netherland; 34 UMC Utrecht Brain Centre, Utrecht University Medical Centre, Utrecht, The Netherlands; 35 Division of Psychological Medicine and Clinical Neurosciences, MRC Centre for Neuropsychiatric Genetics and Genomics, Cardiff University, Cardiff, UK; 36 Department of Psychiatry, Experimental Biomedicine and Clinical Neuroscience, University of Palermo, Palermo, Italy

**Keywords:** polygenic scores, schizophrenia, psychosis, genome-wide association studies, duration of untreated psychosis

## Abstract

Duration of untreated psychosis (DUP) is associated with clinical outcomes in people with a diagnosis of first-episode psychosis (FEP), but factors associated with length of DUP are still poorly understood. Aiming to obtain insights into the possible biological impact on DUP, we report genetic analyses of a large multi-center phenotypically well-defined sample encompassing individuals with a diagnosis of FEP recruited from 6 countries spanning 17 research sites, as part of the European Network of National Schizophrenia Networks Studying Gene-Environment Interactions (EU-GEI) study. Genetic propensity was measured using polygenic scores for schizophrenia (SZ-PGS), bipolar disorder (BD-PGS), major depressive disorder (MDD-PGS), and intelligence (IQ-PGS), which were calculated based on the results from the most recent genome-wide association meta-analyses. Following imputation for missing data and log transformation of DUP to handle skewedness, the association between DUP and polygenic scores (PGS), adjusting for important confounders, was investigated with multivariable linear regression models. The sample comprised 619 individuals with a diagnosis of FEP disorders with a median age at first contact of 29.0 years (interquartile range [IQR] = 22.0–38.0). The median length of DUP in the sample was 10.1 weeks (IQR = 3.8–30.8). One SD increases in SZ-PGS, BD-PGS, MDD-PGS or IQ-PGS were not significantly associated with the length of DUP. Our results suggest that genetic variation does not contribute to the DUP in patients with a diagnosis of FEP disorders.

## Introduction

Despite historical pessimism about schizophrenia prognosis,^1^ it has now been recognized that interventions at the onset of first-episode psychosis (FEP), which is an umbrella term used to refer to schizophrenia spectrum disorders or related psychotic disorders, can improve subsequent illness outcomes.^2,3^ This recognition has led to development of early intervention services, which are founded on an assumption that duration of untreated psychosis (DUP), defined as the time from manifestation of first psychotic symptoms to initiation of adequate treatment,^4^ influences treatment outcomes.^3–5^ Despite the widespread introduction of early intervention services, however, individuals suffering with FEP still experience delays of approximately 1–2 years between onset of first psychotic symptoms and initiation of treatment,^6^ prompting fears of serious consequences on patients’ lives, including enduring deficits and disability.^6^ It is, of course, possible that the relationship between DUP and psychosis outcomes may be a product of other factors^7,8^ related to the organization of mental health system, treatment-seeking behaviors, quality of available treatment,^9^ or poor premorbid functioning. Seen in this way, DUP may be a marker of the illness severity rather than a predictor of the illness itself.

Schizophrenia is a highly heritable disorder, with twin studies estimating its heritability to be more than 75%.^10^ Genomic studies revealed that the genetic architecture of schizophrenia comprises multiple common risk alleles scattered across the whole genome.^11^ Built on the results from the genomic studies, polygenic scores (PGS) analyses confirmed that schizophrenia is highly polygenic nature,^10,11^ where its onset is influenced by many common genetic variants of small effects.^12–14^ Further evidence highlighted that the impact of the combined effect of common genetic markers for schizophrenia, as measured with polygenic score for schizophrenia, extends beyond schizophrenia diagnosis. Indeed, a higher polygenic score for schizophrenia was shown to associate with more severe negative symptoms^15^; whereas, longer DUP is also associated with more severe negative symptoms at first presentation.^16^ Considering negative symptoms were linked to cognitive impairments and deficiencies in social and occupational domains in people with a diagnosis of schizophrenia,^2^ all of which contribute to prolonged delay in seeking help,^17^ it is feasible that length of DUP might be influenced by genetic factors.^8^ However, this question has not been investigated.

Because the etiology of FEP is highly multifactorial, it is likely that other factors may have an important impact on the delay between onset of first psychotic symptoms and initiation of adequate treatment. Certainly, the length of DUP were shown to be influenced by reduced cognitive functioning or intelligence,^18^ severity of depressive symptoms reported in patients with a diagnosis of FEP disorders^19^ and bipolar disorders.^20,21^ Similar to schizophrenia, PGS analyses showed that major depressive disorder, bipolar disorders, and cognition are highly polygenic in nature,^22–24^ with an overlapping, though to varying degree, genetic underpinnings. For example, PGS that combined the additive effect of common genetic markers associated with bipolar disorders discriminated individuals with a diagnosis of schizophrenia^25^ and major depressive disorder from healthy controls.^10,25^ Although much uncertainty remains about their ultimate clinical utility,^26^ PGS have the power to considerably advance our knowledge of the underlying nature of complex phenotypes.^27–29^

Therefore, aiming to obtain insights into possible origins of DUP in people with a diagnosis of FEP disorders, we investigated associations between DUP and PGSs for schizophrenia, bipolar disorders, major depressive disorder, and cognition in a large multi-center phenotypically well-defined sample of individuals with a diagnosis of FEP disorders. Because the length of DUP in individuals with a diagnosis of schizophrenia spectrum disorders is reported to be considerably longer compared to other psychotic disorders,^17,30^ we additionally investigated if our findings were applicable to all individuals with FEP disorders or were specific to patients with first-episode schizophrenia spectrum disorders. We hypothesized that there will be a positive association between polygenic propensity for schizophrenia, bipolar disorder, major depression, and intelligence with longer DUP in participants with a diagnosis of FEP disorders.

## Methods

### Sample

Participants were recruited and assessed as part of the incidence and first episode case-control study, conducted as part of the EUropean network of national schizophrenia networks investigating Gene-Environment Interactions (EU-GEI) study.^31^ EUGEI study was designed to investigate risk factors for psychotic disorders between May 2010 and April 2015 in tightly defined catchment areas in 17 sites across 6 countries, which were the United Kingdom, The Netherlands, France, Spain, Italy, and Brazil.^32^ The research sites within each country were purposefully selected to include a mix of urban and rural areas.^31,32^ The inclusion criteria for FEP cases were: (1) presentation with a clinical diagnosis for a FEP as defined by International Statistical Classification of Diseases and Related Health Problems, 10th Revision (ICD-10) criteria^31^ (codes F20-F33) within the timeframe of the study; (2) aged between 18 and 64 years (inclusive); and (3) resident within one of the 17 defined catchment area at the time of their first presentation to psychiatric services for psychosis. Because the construction of PGSs is dependent on the availability of the summary statistics from genome-wide association studies (GWASs), which are currently based on population of European descent,^33^ for the purpose of the present study we limited participants to those who self-reported to be of European ethnicity. Exclusion criteria were: (1) a previous contact with specialist mental health services for psychotic symptoms outside of the study period at each site; (2) evidence of psychotic symptoms precipitated by an organic cause (ICD-10: F09); (3) transient psychotic symptoms resulting from acute intoxication (F1x.5); (4) severe learning disabilities, defined by an IQ less than 50 or diagnosis of intellectual disability (F70-F79); and (5) insufficient fluency of the primary language at each site to complete assessments.^31^

### Ethical Approval

All participants who agreed to take part in the case-control study provided informed, written consent following full explanation of the study. Ethical approval for the study was provided by relevant local research ethics committees in each of the study sites.^32^

### Assessments

#### Socio-Demographic Characteristics.

Using the Medical Research Council Sociodemographic modified Questionnaire version,^34^ data on socio-demographic characteristics, including gender and country of birth, at the time of the first contact with mental health services for psychosis were collated at each research site. Age at first contact was defined as the age at which a patient was in contact with mental health services for the first time due to their psychotic symptoms. Ethnicity was self-ascribed from the 16 categories employed by the UK Census in 2001 (http://www.statistics.gov.uk/census 2001). Further educational attainment (no qualifications and school qualifications vs higher educational attachment which encompassed tertiary; vocational; undergraduate; postgraduate), employment status (unemployed vs employed [full- or part time] as a reference), living circumstances (currently living with people other than parents vs living alone or/and alone with children) and relationship status (ever vs never in a long-term [>1 year] relationship) were self-reported at first contact with services.

#### *Clinical Measures*.

A modified version of the Nottingham Onset Schedule (NOS)^35^ was used to measure DUP, based on the assessment interview and mental health records, and defined in weeks as the difference between the date of the first positive psychotic symptom (hallucination, delusion or thought disorder- rated as 4 [moderate-severe] or higher on the Positive and Negative Syndrome Scale [PANSS])^36^ and the date of initiation of antipsychotic treatment.^37^ The NOS scale provides a standardized and reliable way of recording early changes in psychosis and identifying relatively precise time points for measuring several durations in emerging psychosis.^35^ The Operational Criteria Checklist (OPCRIT)^38^ systems, whose reliability was assessed before and throughout the study (*k* = 0.7), was used by trained investigators to assess psychopathology in the first 4 weeks after the onset and generate research-based diagnoses based on ICD-10 diagnostic classification systems.^39^ In the present study, diagnoses were grouped using ICD-10 codes into schizophrenia-spectrum disorders (F20-29), bipolar disorder (F30, F31), psychotic depression (F32, F33), and other psychosis.

### Genetic Data

Samples were genotyped at the MRC Centre for Neuropsychiatric Genetics and Genomics in Cardiff (the United Kingdom) using a custom Illumina HumanCoreExome-24 BeadChip genotyping array covering 570038 genetic variants (Illumina Inc.).

#### Quality Control

Quality control (QC) entailed removing samples based on call rate (<0.99), genotype-phenotype mismatched information, suspected non-European ancestry, heterozygosity, and relatedness. Single-nucleotide polymorphism (SNPs) were excluded if the minor allele frequency was 5%, if more than 2% of genotype data were missing and if the Hardy-Weinberg Equilibrium *P*-value < 10^−6^; non-autosomal markers were also removed. The baseline characteristics of participants who were genotyped or were not genotyped are provided in supplementary table 1. To account for any ancestry differences in genetic structures that could bias results, principal components analysis was conducted retaining top principal components (PCs).^40^ Individuals of European ancestry were defined as having PC values within 6 SDs from the mean PC of the EUR in 1000G. Top 20 PCs were retained to adjust for possible population stratification in the association analyses.^40,41^

#### Polygenic Scores

To calculate polygenic score for schizophrenia (SZ-PGS), bipolar disorder (BD-PGS), major depressive disorder (MDD-PGS) and intelligence (IQ-PGS), we used the summary statistics from the latest and largest genome-wide association studies^10,22–24^ utilizing PRSice^42^ where quality-controlled SNPs were pruned using clumping procedure which allowed to obtain SNPs in linkage equilibrium with an *r*^2^ < 0.25 within a 250 kb window. Each PGS was calculated using subsets of the total SNPs based on the *P*-value threshold of .05. The selected *P* value threshold of .05 for SNP inclusion was chosen based on evidence showing that it explains the most variance.^10,22–24^ To aid interpretability of the results, all PGSs were standardized to a mean of 0 (*SD* = 1).

### Statistical Analysis

All analyses reported in the present study were performed using RStudio version 4.0.3.^43^

#### *Imputation of Missing Values*.

In the present study, unemployed (22.9% missing), DUP (15.3% missing), diagnosis (1.8% missing) and living alone (1.0% missing) variable had missing values (supplementary table 2). To avoid using an unrepresentative sample of complete cases that may result in incorrect risk predictions,^44,45^ we conducted an imputation to handle the missing data. To impute the missing values, we employed missForest,^46^ which is an iterative imputation method based on Random Forests. It handles continuous and categorical variables equally well and accommodates non-linear relation structures.^46^ miss-Forest has been shown to outperform the well-known imputation methods, such as *k*-nearest neighbors and parametric multivariate imputation by chained equations.^46^ To evaluate the quality of imputation, we estimated the imputation error Normalized Root Mean Squared Error (NRMSE) for continuous variables and proportion of falsely classified (PFC) for categorical variables.^46,47^ A value close to 0 represents an excellent performance, and a value of 1 indicates poor performance. The imputation of the missing values yielded a minimal error (NRMSE = 0.08%; PFC = 0.13%), highlighting that the imputed values were very closely aligned with the observed values for both continuous and categorical variables. The distribution of the variables included in the analyses before and after the imputation are presented in supplementary table 3.

#### Calculate Power and Predictive Accuracy of PGS

Using information on sample size (*n*), total number of independent markers in genotyping panel (*m*) and lower and upper *P*-values to select markers into polygenic score (*p0, p0.5*) we estimated the predictive accuracy (*R*^2^) present in each PGS employed in the present study using Avengeme package implemented in R.^43^ Consequently, using *n* = 619, and the number of SNPs included in PGS for schizophrenia (*m* = 26 281), bipolar disorder (*m* = 18 092), MDD (*m* = 19 508) and intelligence (*m* = 24 386), we estimated predictive accuracy for each PGS showing that SZ-PGS (*R*^2^ = 0.134, *P* = 7.40 × 10^−23^), BD-PGS (*R*^2^ = 0.005, *P* = .044), MDD-PGS (*R*^2^ = 0.036, *P* = 1.05 × 10^−6^) and IQ-PGS (*R*^2^ = 0.077, *P* = 4.24 × 10^−13^) had sufficient, as indicated by significant *P*-values, predictive accuracy to be employed in the analyses.

#### *Regression Modeling*.

As the frequency distributions of DUP are severely skewed, DUP was normalized by taking the logarithm to base 10 (log_10_DUP) to allow the use of parametric regressions. Following log-transformation, log_10_DUP was normally distributed; distribution of DUP after normalization is presented in supplementary figure 1; the results from the correlations between log_10_DUP and each PGS are provided in supplementary table 5. For each PGS, 2 linear regression models were fitted to understand the role of covariates on the potential relationship of DUP with PGSs: Model 1: crude (unadjusted) model investigating an association between each PGS and DUP; Model 2: Model 1 plus adjusting for age at first contact with mental health services for psychosis, gender, genetic ancestry as measured with first 4 PCs, research sites and educational attainment. To measure prediction accuracy of each PGS, we utilized the incremental *R*^2^, which was calculated following the previously outlined steps.^48^ Specifically, to calculate *R*^2^ value for each model, we first regressed a phenotype on our set of controls without the PGS; we then re-ran the same regression but with the PGS included as a regressor.

#### *Sensitivity Analyses*.

To examine whether our findings were applicable to individuals with a diagnosis of FEP disorders or were specific to people with a diagnosis of first-episode schizophrenia spectrum disorders only, we repeated the analyses limiting them to those who received the diagnosis of schizophrenia spectrum disorders on the first contact with mental health services. We further investigated if the results would remain the same using unimputed (complete cases) variables. As this was an exploratory study, which does not strictly require adjustment for multiple comparisons,^49^ we did not employ correction for multiple testing. All tests for analyses were 2-tailed; *P*-values ≤ .05 were considered statistically significant.

## Results

### Sample Characteristics

The demographic characteristics of the analytic sample of FEP cases are presented in table 1. The sample comprised 619 (86.6% of *N* = 715) individuals of European ancestry for whom quality-controlled genome-wide genotyping and DUP were available. Those participants who were included in the study or excluded from the final cohort did not differ in terms of DUP, gender, marital status, employment, living arrangement, and diagnoses; however, the former group included participants who were younger (*t*_(1112.5)_ = −2.31, *P* = .021) and had a lower educational attainment (x^2^_(1)_ = 4.72, *P* = .030) compared to those who were included in the study (supplementary table 4). The median age at first contact was 29.0 years (IQR = 22.0–38.0). Of the entire sample, 37.3% (*N* = 227/669) had diagnoses of first-episode schizophrenia spectrum disorders, 63.6% (*N* = 394) were men, 37.3% (*N* = 178) were unemployed and 18.8% lived alone at the time of the first contact with mental health services for psychosis.

**Table 1.** Baseline Sociodemographic and Clinical Characteristics of First-Presentation Psychosis Patients

Baseline Sample Characteristics	Total Sample (*N* = 619)
	*N* (%)/mean (SD)
Age (y)	31.5 (10.9)
DUP (wk)	62.5 (191.6)
Male gender	394 (63.6)
Not married	444 (72.1)
Unemployed	178 (37.3)
Living alone	115 (18.8)
Low educational attainment	88 (14.3)
Diagnosis	
Schizophrenia spectrum	250 (11.0)
Bipolar Disorder	67 (11.0)
Psychotic depression	74 (12.2)
Other psychosis	217 (35.7)
Country of data collection	
The United Kingdom	99 (16.0)
Holland	133 (21.5)
Spain	151 (24.4)
France	24 (6.8)
Italy	103 (16.6)
Brazil	91 (14.7)

*Note:* DUP, duration of untreated psychosis.

### Length of DUP by European Countries and FEP Diagnoses

The median length of DUP in the whole sample was 10.0 weeks (interquartile range [IQR] = 3.8–30.8). DUP did not differ by countries: France (median = 12.3 weeks, IQR = 80.8), the United Kingdom (median = 10.9 weeks, IQR = 2.42–51.93), The Netherlands (median = 10.3 weeks, IQR = 3.42–29.63), Italy (median = 8.1 weeks, IQR = 3.85–18.80), Spain (median = 8.7 weeks, IQR = 4.27–34.83), and Brazil (median = 9.4 weeks, IQR = 4.13–24.08) (Kruskal-Wallis_(6)_ = 4.61, *P* = .595). When DUP was stratified by diagnoses, the longest DUP was observed in patients with diagnosis of schizophrenia spectrum (median = 20.8 weeks, IQR = 10.11–80.07) followed by psychotic depression (median = 11.5 weeks, IQR = 6.41–22.65) and bipolar disorders (median = 4.42 weeks, IQR = 1.71–8.69) (Kruskal-Wallis_(3)_ = 110.0, *P* = 1.09 × 10^−23^).

### Associations Between PGSs and DUP

Associations between length of DUP and PGS in patients with FEP are presented in table 2. One SD increase in SZ-PGS was not significantly associated with the length of DUP in participants with a diagnosis of FEP disorders (Model 2: *β*_adjusted_ = −0.11, 95% CI = −0.341–0.131, *R*^2^ = 0.023). Similarly, there were no statistically significant associations between DUP and BD-PGS (Model 2: *β*_adjusted_ = 0.050, 95% CI = −0.123–0.223, *R*^2^ = 0.022), MDD-PGS (Model 2: *β*_adjusted_ = 0.036, 95% CI = −0.094–0.167, *R*^2^ = 0.022) or IQ-PGS (Model 2: *β*_adjusted_ = −0.017, 95% CI = −0.160–0.125, *R*^2^ = 0.022) in participants with a diagnosis of FEP disorders. These results did not differ from those observed in Model 1 (ie, the unadjusted model) and when complete-case (unimputed) data were employed to run the models (supplementary table 6). When analyses were limited to participants with a diagnosis of first-episode schizophrenia spectrum, we did not find significant associations between each polygenic score and DUP in unadjusted and fully adjusted models (supplementary table 7).

**Table 2.** Associations Between Length of Untreated Psychosis and PGS in Patients With FEP

PGS	Model 1			Model 2		
	β (95% CI)	*P*-Value	Model Fit	β (95% CI)	*P*-Value	Model Fit
SZ-PGS	0.052 (−0.089–0.194)	.468	*R*^2^ = 0.001	−0.110 (−0.341–0.131)	.467	*R*^2^ = 0.023
BD-PGS	−0.020 (−0.161–0.122)	.785	*R*^2^ = 0.000	0.050 (−0.123–0.223)	.389	*R*^2^ = 0.022
MDD-PGS	0.060 (−0.081–0.201)	.405	*R*^2^ = 0.001	0.036 (−0.094–0.167)	.149	*R*^2^ = 0.022
IQ-PGS	0.008 (−0.133–0.150)	.907	*R*^2^ = 0.000	−0.017 (−0.160–0.125)	.776	*R*^2^ = 0.022

*Note:* Effect size is indicated by β coefficient from the linear regression model; the presented β coefficient is standardized. CI, confidence interval; SZ-PGS, polygenic score for schizophrenia; BD-PGS, bipolar disorders; MDD-PGS, major depressive disorder; IQ-PGS, intelligence; PGS, polygenic scores; FEP, first episode psychosis. Model 1: crude (unadjusted) model investigating an association between each PGS and DUP; Model 2: Model 1 plus adjusting for age at first contact with mental health services for psychosis, gender, genetic ancestry as measured with first 4 principal components, research site and educational attainment.

## Discussion

To our knowledge, this is the first study investigating the relationship of polygenic propensity for schizophrenia, bipolar disorder, major depressive disorder, and intelligence with duration of untreated psychosis.

Consistent with previous reports,^50–52^ our findings showed that individuals with a diagnosis of FEP disorders had to endure a prolonged period coping with symptoms of psychosis without seeking appropriate treatments; though, this was heavily skewed with a smaller subset of participants experiencing over a year before first contact with mental health services. Similar to previous reports,^17,30^ we observed that the median length of DUP in participants with schizophrenia spectrum disorders was significantly longer when compared to all other psychoses. These observed delays highlight that there is still a great need to improve recognition of the symptoms of FEP, including schizophrenia and pathways to care.

The neurodevelopmental theory of schizophrenia posits that genetic factors interfere with early brain development leading to the development of schizophrenia symptoms.^53^ This, in combination of accumulated evidence for polygenicity of schizophrenia,^10^ alluded to a possibility that the length of DUP might also be influenced by additive effect of multiple common genetic markers linked to schizophrenia.^8^ However, this hypothesis was not confirmed by our findings. We further considered that high genetic predisposition for either bipolar disorder or major depression disorder might be associated with DUP in people with a diagnosis of FEP disorders. Once again, our findings were negative, as they were for PGS for intelligence. It may be argued that a limited power might have led to these non-significant results. Because the PGS employed in this study were built using the results from most recent and largest GWAS meta-analyses, our analyses were not constrained by our sample size.^43,54^ Nonetheless, to ensure we captured the true polygenic contribution to DUP, we undertook calculations of power for each polygenic score, which revealed that there was considerable predicative power in each PGS to detect potential associations. Our results are further in line with a recent literature review highlighting that evidence of an association between DUP and brain structure in people with a diagnosis FEP disorders was minimal.^55^ It is further argued that any relationships observed between untreated psychosis and psychosis illness course appears to be explained by lead-time bias.^7^ Accordingly, those with a short DUP are in an earlier stage and therefore are likely to have better outcomes than those with a long DUP, who are in a later stage.^7^ Cumulatively, our findings shed some doubt on the notion that genetic variation has substantial impact on DUP.^56^

In light of these findings, a discussion of some alternative theories explaining the length of DUP is warranted. It has been suggested that the length of delay from first manifestation of psychotic symptoms to initiation of adequate treatment may be influenced by factors related to the organization of mental health system and process of referral to an appropriate service FEP. Reduced allocated resources for early intervention services^57^ and limited availability of care^58^ may also be important contributing factors to longer DUP. The lack of knowledge of what constitutes psychosis onset^5,37^and what help may be available for people affected by early psychosis and their families^59^ were shown to be important factors influencing DUP.^5,37^ The longer delays to seeking help for first-episode psychotic disorders were further linked fear of stigma. Therefore, DUP may be significantly reduced through educational and anti-stigmatizing campaign about the signs of early psychosis targeted at health care providers, public, and schools increasing the motivation to seek treatment.^58^ Although evidences regarding successfulness of specific interventions in reducing DUP are still lacking and largely non replicated,^60^ our findings should encourage the identification of potentially effective initiatives.

### Methodological Strengths and Limitations

This is an extensive multi-site study of FEP with comprehensive data on a variety of environmental and genetic factors. The study included all incidence cases from well-defined catchment areas in 17 sites across 6 countries. As our analyses were focused on people with a diagnosis of FEP, the findings reported in the present study are less likely to be biased toward patients who experience multiple hospital admissions.^61,62^ Given that our study was carried out in major urban and rural sites with heterogeneous populations suggest that the generalizability of our findings may extend to other centers with similar population profiles. Finally, because the calculation of PGS is based on well-powered GWASs, we did not require a large sample to test our hypotheses, which was further confirmed by estimated predictive power in each PGS employed in the analyses.

Nonetheless, important methodological considerations warrant a discussion. While it is likely most individuals who develop a psychotic disorder do present to services, at least in sites with well-developed public health systems, some who do not present will be missed and this may introduce selection biases.^31^ Variations in referral procedures of patients with psychosis from primary to secondary mental health care settings and in the organization of secondary mental health care services across catchment areas may have influenced the identification of cases.^31^ Even though robust imputation methods have been employed to deal with missing values, the percentage of missingness in our variables, though lower than previously, is a notable issue in the present study. Although the median age of our sample was consistent with that of other very large samples of individuals with FEP collated in Europe, Australia and some studies from North American,^63–65^ it may still be higher compared to other studies from the US. Thus, we urge caution when generalizing our findings to all patients with FEP across the continents. It may be argued that the length of DUP observed in our study was shorter than that reported in some other studies,^50–52^ which in turn may have reduced the likelihood of finding a significant association with PGS. The poor generalizability of genetic studies across populations is also noteworthy.^33^ This is because the construction of PGSs is largely dependent on the availability of the summary statistics from genome-wide association studies (GWASs). However, around 79% of all GWAS participants are of European descent despite making up only 16% of the global population.^33^ Given genetic risk is different in European and non-European individuals, further work is necessary to develop PGSs model in non-White populations. Because our analyses were restricted to individuals of European ethnicity, our results do not shed light on the associations of genetic predisposition to the major psychiatric conditions and DUP among people of non-European ethnicity. This is an important limitation as Black-African and Black-Caribbean groups were shown to have a significantly different length of DUP relative to White groups,^66^ which in turn may reflect differences in pathways to care experienced by some ethnic minority groups.^67^ Finally, by their design, PGSs do not capture other structural variants beyond common genetic markers of relatively small effects, such as rare variants, poorly tagged or multiple independent variants, gene-by-gene interaction and gene-by-environment interplay.^68^

## Conclusion

Although our findings are specific to individuals from European populations, our results suggest there are not strong genetic risk factors underlying duration of untreated psychosis, underscoring the importance of effective educational efforts directed towards the public, the schools, and the health professionals about first onset of psychotic disorders.

## Supplementary Material

sbab055_suppl_Supplementary-Tables

## Notes

*EU-GEI Collaborators*: Hubbard Kathryn^1^, Beards Stephanie^1^, Stilo Simona A.^2^, Parellada Mara^3^, Cuadrado Pedro^4^, Solano Jose Juan Rodriguez^5^, David Fraguas^5^, Andreu-Bernabeu Alvaro^5^, Carracedo Angel^6^, Bernardo Enrique Garcia^7^, Roldan Laura^3^, Lopez Gonzalo^3^, Cabrera Bibiana^8^, Nacher Juan^9^, Garcia-Portilla Paz^10^, Costas Javier^6^, Jimenez-Lopez Estela^11^, Matteis Mario^3^, Castro Marta Rapado^3^, Gonzalez Emiliano^3^, Martinez Covadonga^3^, Sanchez Emilio^7^, Duran-Cutilla Manue^l7^, Franke Nathalie^12^, Termorshuizen Fabian^13^, ^14^, van Dam Daniella^12^, van der Ven Elsje^13,14^, Messchaart Elles^14^, Leboyer Marion^15,16,17,18^, Schu.rhoff Franck^15,16,17,18^, Jamain Stephane^16,17,18^, Baudin Gregoire^15,16^, Ferchiou Aziz^15,16^, Pignon Baptiste^15,16,18^, Richard Jean- Romain^16,18^, Charpeaud Thomas ^18,19,21^, Tronche Anne-Marie^18,19,21^, Frijda Flora^22^, La Barbera Daniele^22,23^, Marrazzo Giovanna^23^, Sideli Lucia^22^, Sartorio Crocettarachele^22,23^, Ferraro Laura^22^,Seminerio Fabio^22^, Loureiro Camila Marcelino^24,25^, Shuhama Rosana^24,25^, Ruggeri Mirella^26^, LaSalvia Antonio^26^, Bonetto Chiara^26^, and Cristofalo Doriana26. Corresponding affiliations: ^1^Department of Health Service and Population Research, Institute of Psychiatry, King’s College London, De Crespigny Park, Denmark Hill, London, United Kingdom, SE5 8AF; ^2^Department of Psychosis Studies, Institute of Psychiatry, King’s College London, De Crespigny Park, Denmark Hill, London, United Kingdom SE5 8AF; ^3^Department of Child and Adolescent Psychiatry, Hospital General Universitario Gregorio Marañón, School of Medicine, Universidad Complutense, IiSGM (CIBERSAM), C/Doctor Esquerdo 46, 28007 Madrid, Spain; ^4^Villa de Vallecas Mental Health Department, Villa de Vallecas Mental Health Centre, Hospital Universitario Infanta Leonor / Hospital Virgen de la Torre, C/San Claudio 154, 28038 Madrid, Spain; ^5^Puente de Vallecas Mental Health Department, Hospital Universitario Infanta Leonor / Hospital Virgen de la Torre, Centro de Salud Mental Puente de Vallecas, C/Peña Gorbea 4, 28018 Madrid, Spain; ^6^Fundación Pública Galega de Medicina Xenómica, Hospital Clínico Universitario, Choupana s/n, 15782 Santiago de Compostela, Spain; ^7^Department of Psychiatry, Hospital General Universitario Gregorio Marañón, School of Medicine, Universidad Complutense, IiSGM (CIBERSAM), C/Doctor Esquerdo 46, 28007 Madrid, Spain; ^8^Department of Psychiatry, Hospital Clinic, IDIBAPS, Centro de Investigación Biomédica en Red de Salud Mental (CIBERSAM), Universidad de Barcelona, C/Villarroel 170, escalera 9, planta 6, 08036 Barcelona, Spain; ^9^Neurobiology Unit, Program in Neurosciences and Interdisciplinary Research Structure for Biotechnology and Biomedicine (BIOTECMED), Universitat de València, Burjassot, Spain Biomedical Research Networking Centre in Mental Health (CIBERSAM), Madrid, Spain. Biomedical Research Institute INCLIVA, Valencia, Spain; ^10^Department of Medicine, Psychiatry Area, School of Medicine, Universidad de Oviedo, Centro de Investigación Biomédica en Red de Salud Mental (CIBERSAM), C/Julián Clavería s/n, 33006 Oviedo, Spain; ^11^Department of Psychiatry, Servicio de Psiquiatría Hospital “Virgen de la Luz,” C/Hermandad de Donantes de Sangre, 16002 Cuenca, Spain; ^12^Department of Psychiatry, Early Psychosis Section, Academic Medical Centre, University of Amsterdam, Meibergdreef 5, 1105 AZ Amsterdam, The Netherlands; ^13^Rivierduinen Centre for Mental Health, Leiden, Sandifortdreef 19, 2333 ZZ Leiden, The Netherlands; ^14^Department of Psychiatry and Neuropsychology, School for Mental Health and Neuroscience, South Limburg Mental Health Research and Teaching Network, Maastricht University Medical Centre, P.O. Box 616, 6200 MD Maastricht, The Netherlands; ^15^AP-HP, Groupe Hospitalier “Mondor,” Pôle de Psychiatrie, 51 Avenue de Maréchal de Lattre de Tassigny, 94010 Créteil, France; ^16^INSERM, U955, Equipe 15, 51 Avenue de Maréchal de Lattre de Tassigny, 94010 Créteil, France; ^17^Faculté de Médecine, Université Paris-Est, 51 Avenue de Maréchal de Lattre de Tassigny, 94010 Créteil, France; ^18^Fondation Fondamental, 40 Rue de Mesly, 94000 Créteil, France; ^19^CMP B CHU, BP 69, 63003 Clermont Ferrand, Cedex 1, France; ^20^ EPS Maison Blanche, Paris 75020, France; ^21^ Université Clermont Auvergne, EA 7280, Clermont-Ferrand, 63000, France; ^22^Department of Experimental Biomedicine and Clinical Neuroscience, Section of Psychiatry, University of Palermo, Via G. La Loggia n.1, 90129 Palermo, Italy; ^23^Unit of Psychiatry, “P. Giaccone” General Hospital, Via G. La Loggia n.1, 90129 Palermo, Italy; ^24^Departamento de Neurociências e Ciencias do Comportamento, Faculdade de Medicina de Ribeirão Preto, Universidade de São Paulo, Av. Bandeirantes, 3900 - Monte Alegre- CEP 14049–900, Ribeirão Preto, SP, Brasil; ^25^Núcleo de Pesquina em Saúde Mental Populacional, Universidade de São Paulo, Avenida Doutor Arnaldo 455, CEP 01246-903, SP, Brasil; ^26^Section of Psychiatry, Department of Neuroscience, Biomedicine and Movement, University of Verona, Piazzale L.A. Scuro 10, 37134 Verona, Italy.
