# Supplementary material for: Duration of Untreated Psychosis in First-Episode Psychosis is not Associated With Common Genetic Variants for Major Psychiatric Conditions: Results From the Multi-Center EU-GEI Study
Source: Schizophr Bull. 2021 May 8;47(6):1653–62. doi: 10.1093/schbul/sbab055 (PMC8562562; doi:10.1093/schbul/sbab055)

**Supplementary Table 1. The baseline characteristics of EIGEI participants who were either genotyped or were not genotyped**

| **Baseline characteristics** | | |  | **GWAS - NO**  **N=556**  **(N_FEP_=282, N_controls_=274)** | | **GWAS - YES**  **N=2,071**  **(N_FEP_=856,N_controls_=1215)** | **Test statistics** | | | | |
| --- | --- | --- | --- | --- | --- | --- | --- | --- | --- | --- | --- |
|  |  | |  | **Mean (SD)/N(%)** | | **Mean (SD)/N(%)** | **t/x^2^** | **df** | | **P-value** | |
|  | | |  |  | |  |  |  | |  | |
| Age (years) | | |  | 32.6 (11.5) | | 34.3 (12.4) | 2.9 | 2,2642 | | .05 | |
|  |  | |  |  | |  |  |  | |  | |
| Gender (Male) | | |  | 299 (53.8) | | 1,104 (53.3) | 0.03 | 1 | | .84 | |
|  |  | |  |  | |  |  |  | |  | |
| Self-reported Ethnicity | | |  | |  |  |  | | 5 | | .13 |
|  | | White |  | | 374 (67.3) | 1,520 (73.4) |  | |  | |  |
|  | | Black |  | | 78 (14.0) | 226 (10.9) |  | |  | |  |
|  | | Mixed |  | | 54 (9.7) | 172 (8.3) |  | |  | |  |
|  | | Asian |  | | 17 (3.1) | 51 (2.5) |  | |  | |  |
|  | | North African |  | | 19 (3.4) | 57 (2.7) |  | |  | |  |
|  | | Other |  | | 14 (2.5) | 45 (2.2) |  | |  | |  |
|  | |  |  | |  |  |  | |  | |  |
| Country | | |  | |  |  | 78.7 | | 5 | | .001 |
|  | | United Kingdom |  | | 123 (22.1) | 459 (22.2) |  | |  | |  |
|  | | Holland |  | | 54 (9.7) | 352 (17.0) |  | |  | |  |
|  | | Spain |  | | 74 (13.3) | 352 (17.0) |  | |  | |  |
|  | | France |  | | 71 (12.8) | 181 (8.7) |  | |  | |  |
|  | | Italy |  | | 157 (28.2) | 310 (15.0) |  | |  | |  |
|  | | Brazil |  | | 77 (13.8) | 417 (20.1) |  | |  | |  |
|  | |  |  | |  |  |  | |  | |  |
| Research Domain Criteria Diagnosis | | |  | |  |  | 3.3 | | 4 | | .500 |
|  | | Bipolar disorder |  | | 13 (4.7) | 47 (5.5) |  | |  | |  |
|  | | Major depression with psychotic features |  | | 18 (5.9) | 32 (4.0) |  | |  | |  |
|  | | Schizophrenia |  | | 84 (30.7) | 306 (35.7) |  | |  | |  |
|  | | Schizoaffective disorder |  | | 116 (42.3) | 318 (37.1) |  | |  | |  |
|  | | Unspecified psychosis |  | | 48 (17.5) | 148 (17.3) |  | |  | |  |

df, degree of freedom; SD standard deviation;

**Supplementary Table 2. Distribution of missing and observed variables included in the analyses**

|  |  | **N**  **missing** | **%**  **missing** | **N**  **complete** | **%**  **complete** |
| --- | --- | --- | --- | --- | --- |
|  |  |  |  |  |  |
| Age years |  | 0 | 0.0 | 619 | 100.0 |
|  |  |  |  |  |  |
| DUP weeks |  | 95 | 15.35 | 524 | 84.6 |
|  |  |  |  |  |  |
| Male gender |  | 0 | 0.0 | 619 | 100.0 |
|  |  |  |  |  |  |
| Not married |  | 3 | 0.48 | 616 | 99.5 |
|  |  |  |  |  |  |
| Unemployed |  | 142 | 22.94 | 477 | 77.1 |
|  |  |  |  |  |  |
| Low educational attainment |  | 0 | 0 | 619 | 100.0 |
|  |  |  |  |  |  |
| Living alone |  | 6 | 0.97 | 610 | 99.0 |
|  |  |  |  |  |  |
| Diagnosis |  | 11 | 1.78 | 608 | 98.2 |
|  |  |  |  |  |  |
| Country of data collection |  | 0 | 0.0 | 619 | 100.0 |

**Supplementary Table 3. Distributions of the variables before and after imputation**

|  | **Variables at baseline** |  | **Variables before imputation** | **Variables after imputation** |
| --- | --- | --- | --- | --- |
|  |  |  | **Mean (SD) / N (%)** | **Mean (SD) / N (%)** |
| Age years | |  | 31.5 (10.9) | 31.5 (10.9) |
|  |  |  |  |  |
| DUP weeks | |  | 62.5 (191.6) | 55.7 (177.1) |
|  |  |  |  |  |
| Male gender | |  | 394 (63.6) | 394 (63.6) |
|  |  |  |  |  |
| Not married | |  | 444 (72.1) | 445 (71.9) |
|  |  |  |  |  |
| Unemployed | |  | 178 (37.3) | 269 (43.5) |
|  | |  |  |  |
| Low educational attainment | |  | 88 (14.3) | 88 (14.3) |
|  |  |  |  |  |
| Living alone | |  | 115 (18.8) | 115 (18.6) |
|  |  |  |  |  |
| Schizophrenia diagnosis | |  | 131 (21.9) | 133 (21.5) |
|  |  |  |  |  |
| Country of data collection | |  |  |  |
|  | UK |  | 99 (16.0) | 99 (16.0) |
|  | Holland |  | 133 (21.5) | 133 (21.5) |
|  | Spain |  | 151 (24.4) | 151 (24.4) |
|  | France |  | 24 (6.8) | 24 (6.8) |
|  | Italy |  | 103 (16.6) | 103 (16.6) |
|  | Brazil |  | 91 (14.7) | 91 (14.7) |

SD, standard deviation

**Supplementary Table 4. The baseline characteristics of individuals with first episode psychosis who were either included in the present study or excluded from the study**

| **Baseline characteristics** | | **Excluded**  **N=511** | **Included**  **N=619** | **Statistics** |
| --- | --- | --- | --- | --- |
|  |  | **Mean (SD)/N(%)** | **Mean (SD)/N(%)** | **t/x^2^ (df), P-value** |
| Age years | | 30.0 (10.2) | 31.5 (10.9) | -2.31 (1112.5), .021 |
|  |  |  |  |  |
| DUP weeks | | 9.4 (40.0) | 10.1 (26.9) | 0.29 (1), .587 |
|  |  |  |  |  |
| Male gender | | 303 (43.5) | 394 (56.5) | 2.25 (1), .134 |
|  |  |  |  |  |
| Not married | | 354 (44.4) | 444 (55.6) | 0.53 (1), .467 |
|  |  |  |  |  |
| Unemployed | | 190 (51.6) | 178 (48.4) | 2.87 (1), .09 |
|  |  |  |  |  |
| Living alone | | 109 (48.6) | 115 (51.3) | 1.50 (1), .221 |
|  |  |  |  |  |
| Diagnosis | |  |  | 2.54, (3), .468 |
|  | Other psychosis | 180 (45.3) | 217 (54.6) |  |
|  | Psychotic depression | 47 (38.8) | 74 (61.1) |  |
|  | Bipolar disorders | 63 (48.5) | 67 (51.5) |  |
|  | Schizophrenia spectrum | 181 (44.1) | 227 (55.9) |  |
|  |  |  |  |  |
| Low educational attainment | | 97 (52.4) | 88 (47.6) | 4.72 (1), .030 |
|  |  |  |  |  |
| Country of data collection | |  |  | 78.49 (5), 1.74e^-15^ |
|  | UK | 147 (59.8) | 99 (40.2) |  |
|  | Holland | 63 (32.1) | 133 (67.9) |  |
|  | Spain | 53 (26.0) | 151 (74.0) |  |
|  | France | 63 (60.0) | 42 (40.0) |  |
|  | Italy | 84 (44.9) | 103 (55.1) |  |
|  | Brazil | 101 (52.6) | 91 (47.4) |  |

df, degree of freedom; SD standard deviation;

**Supplementary Table 5**. Correlations between log-transformed duration of untreated psychosis (log_10_DUP) and polygenic scores

| Polygenic scores |  | log_10_DUP | |
| --- | --- | --- | --- |
|  |  | Corelation | P-value |
| SZ-PGS |  | 0.029 | .468 |
| BD-PGS |  | -0.011 | .784 |
| MDD-PGS |  | 0.033 | .405 |
| IQ-PGS |  | 0.005 | .907 |

SZ-PGS, polygenic score for schizophrenia; BD-PGS, bipolar disorders; MDD-PGS, major depressive disorder; IQ-PGS, intelligence.

**Supplementary Table 6**. **Associations between length of untreated psychosis and polygenic scores in patients with first episode psychosis using unimputed (i.e., complete cases) data**

| **Polygenic scores** | **Model 1** | | |  | **Model 2** | | | |  |
| --- | --- | --- | --- | --- | --- | --- | --- | --- | --- |
|  | β (95%CI) | P-value | Model fit |  | | β (95%CI) | P-value | Model fit | |
|  |  |  |  |  | |  |  |  | |
| SZ-PGS | 0.047 (-0.118-0.212) | 0.576 | *R*²=0.001 |  | | -0.118 (-0.401-0.166) | 0.416 | *R*²=0.028 | |
| BD-PGS | -0.023 (-0.188-0.143) | 0.788 | *R*²=0.000 |  | | 0.120 (-0.087-0.328) | 0.255 | *R*²=0.029 | |
| MDD-PGS | 0.086 (-0.079-0.251) | 0.305 | *R*²=0.002 |  | | 0.093 (-0.061-0.247) | 0.236 | *R*²=0.029 | |
| IQ-PGS | 0.015 (-0.150-0.180) | 0.859 | *R*²=0.000 |  | | -0.018 (-0.183-0.147) | 0.831 | *R*²=0.027 | |

Effect size is indicated by β coefficient from the linear regression model; the presented β coefficient is standardised

CI, confidence interval; SZ-PGS, polygenic score for schizophrenia; BD-PGS, bipolar disorders; MDD-PGS, major depressive disorder; IQ-PGS, intelligence.

Model 1: crude (unadjusted) model investigating an association between each PGS and DUP; Model 2: Model 1 plus adjusting for age at first contact with mental health services for psychosis, gender, genetic ancestry as measured with first four principal components, research site and educational attainment.

**Supplementary Table 7**. **Associations between length of untreated psychosis and polygenic scores in patients with first episode schizophrenia**

| **Polygenic scores** | **Model 1** | | | |  | **Model 2** | | | |  |
| --- | --- | --- | --- | --- | --- | --- | --- | --- | --- | --- |
|  | β (95%CI) |  | Model fit |  | | | β (95%CI) |  | Model fit | |
|  |  |  |  |  | | |  |  |  | |
| SZ-PGS | -0.012 (-0.183-0.159) | 0.436 | *R*²=0.000 |  | | | -0.039 (-0.362-0.283) | 0.635 | R² = 0.051 | |
| BD-PGS | 0.088 (-0.082-0.259) | 0.694 | *R*²=0.004 |  | | | 0.176 (-0.053-0.405) | 0.728 | R² = 0.059 | |
| MDD-PGS | 0.061 (-0.126-0.248) | 0.665 | *R*²=0.002 |  | | | 0.076 (-0.115-0.267) | 0.727 | R² = 0.053 | |
| IQ-PGS | 0.011 (-0.187-0.209) | 0.153 | *R*²=0.000 |  | | | 0.040 (-0.176-0.256) | 0.192 | R² = 0.051 | |

Effect size is indicated by β coefficient from the linear regression model; Effect size is indicated by β coefficient from the linear regression model; the presented β coefficient is standardised

CI, confidence interval; SZ-PGS, polygenic score for schizophrenia; BD-PGS, bipolar disorder; MDD-PGS, major depressive disorder; IQ-PGS, intelligence.

Model 1: crude (unadjusted) model investigating an association between each PGS and DUP; Model 2: Model 1 plus adjusting for age at first contact with mental health services for psychosis, gender, genetic ancestry as measured with 4 principal components, diagnosis, research site and educational attainment.

**Supplementary Figure 1. Distribution of duration of untreated psychosis post log-transformation**


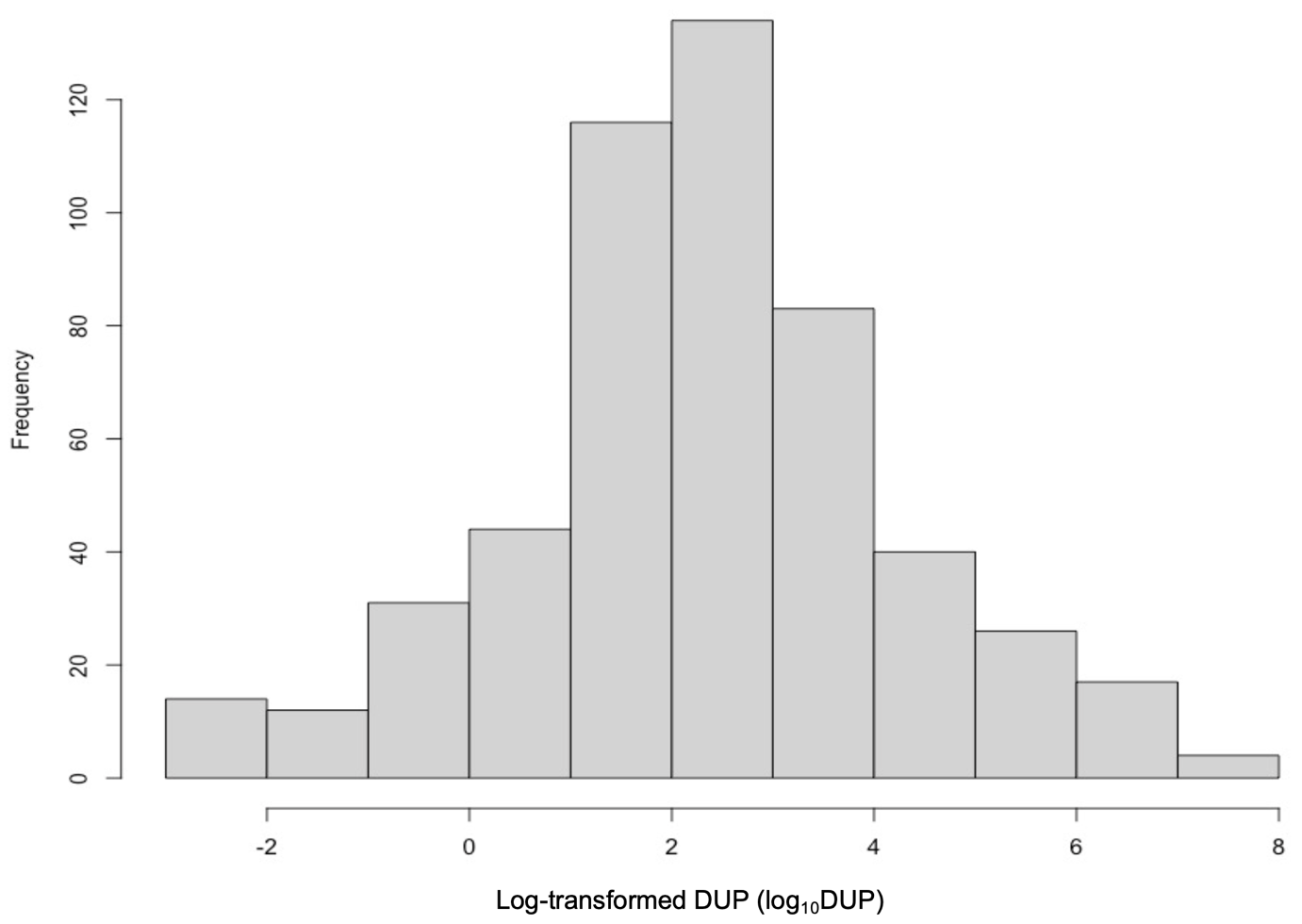

Supplement: sbab055_suppl_Supplementary-Tables [file sbab055_suppl_supplementary-tables.docx]
